# Current Status and Future Prospects of Genome-Scale Metabolic Modeling to Optimize the Use of Mesenchymal Stem Cells in Regenerative Medicine

**DOI:** 10.3389/fbioe.2020.00239

**Published:** 2020-03-31

**Authors:** Þóra Sigmarsdóttir, Sarah McGarrity, Óttar Rolfsson, James T. Yurkovich, Ólafur E. Sigurjónsson

**Affiliations:** ^1^The Blood Bank, Landspitali – The National University Hospital of Iceland, Reykjavik, Iceland; ^2^School of Science and Engineering, Reykjavik University, Reykjavik, Iceland; ^3^Faculty of Medicine, School of Health Sciences, University of Iceland, Reykjavik, Iceland; ^4^Institute for Systems Biology, Seattle, WA, United States

**Keywords:** MSCs, metabolism, personalized/precision medicine, metabolomics, metabolic modeling, tissue engineering

## Abstract

Mesenchymal stem cells are a promising source for externally grown tissue replacements and patient-specific immunomodulatory treatments. This promise has not yet been fulfilled in part due to production scaling issues and the need to maintain the correct phenotype after re-implantation. One aspect of extracorporeal growth that may be manipulated to optimize cell growth and differentiation is metabolism. The metabolism of MSCs changes during and in response to differentiation and immunomodulatory changes. MSC metabolism may be linked to functional differences but how this occurs and influences MSC function remains unclear. Understanding how MSC metabolism relates to cell function is however important as metabolite availability and environmental circumstances in the body may affect the success of implantation. Genome-scale constraint based metabolic modeling can be used as a tool to fill gaps in knowledge of MSC metabolism, acting as a framework to integrate and understand various data types (e.g., genomic, transcriptomic and metabolomic). These approaches have long been used to optimize the growth and productivity of bacterial production systems and are being increasingly used to provide insights into human health research. Production of tissue for implantation using MSCs requires both optimized production of cell mass and the understanding of the patient and phenotype specific metabolic situation. This review considers the current knowledge of MSC metabolism and how it may be optimized along with the current and future uses of genome scale constraint based metabolic modeling to further this aim.

## Introduction

In recent years, there has been increasing interest in the possibilities offered by regenerative medicine (Maienschein, 2011; Sampogna et al., 2015), a field which seeks solutions for the restoration of the structure and functions of organs and tissues that have become permanently damaged. While regenerative medicine has enjoyed success in some areas, treatment can result in danger to patients or in therapeutic inefficiency (Neman et al., 2012; Campana et al., 2014; Goldberg et al., 2017; Moreira et al., 2017; Cunningham et al., 2018; Solarte et al., 2018) that have pushed researchers to continuously search for novel approaches to address limitations.

One important area of regenerative medicine is the use of stem cells to enhance available therapeutic applications and to further the development of new ones (Mahla, 2016). In particular, mesenchymal stem cells, or mesenchymal stromal cells (MSCs) (Rosenbaum et al., 2008; Ullah et al., 2015; Fitzsimmons et al., 2018), are of interest. MSCs are multipotent cell types with stem cell-like abilities that can be isolated from various adult and neonatal tissues (Nombela-Arrieta et al., 2011; Alberts et al., 2014). MSCs maintain proliferation abilities while possessing the ability to undergo trilineage differentiation (adipogenic, chondrogenic, and osteogenic differentiation) and remarkable immunomodulatory capabilities (Rosenbaum et al., 2008; Lin et al., 2013). These properties offer the possibility of furthering treatment options for various ailments, such as metabolic and autoimmune diseases like multiple sclerosis (Bonab et al., 2012), Alzheimer’s disease (Rosenbaum et al., 2008; Mahla, 2016), diabetes (Ullah et al., 2015; Fitzsimmons et al., 2018), Crohn’s disease (Duijvestein et al., 2010), and cancer (Qin et al., 2016).

For the past decade, MSCs metabolism has received growing interest due to mounting evidence suggesting that the manipulation of metabolism allows enhanced therapeutic uses of these cells (e.g., cell retention, cell survival, immunoregulation, differentiation) in cell-based medicine and tissue engineering (Chen et al., 2008; Croitoru-Lamoury et al., 2011; Pattappa et al., 2011; Buravkova et al., 2013; Beegle et al., 2015; Shum et al., 2016; Li et al., 2017; Meyer et al., 2018; Vigo et al., 2019; Zhu and Thompson, 2019). Cellular metabolism is an intricate and complex network of pathways, enzymatic reactions, metabolites, and co-factors with numerous effects on the cell and its immediate surroundings. Due to the complexity of metabolism and its effects, research into the possibilities of manipulating metabolism in these cells has been slow.

The advent of high-throughput -omic technologies (Henry et al., 2010; Resendis-Antonio, 2013) has allowed for the detailing of genome-scale metabolic networks (Yurkovich and Palsson, 2016). This holistic systems biology approach acknowledges that biological systems are made up of a network of networks (Reed and Palsson, 2003; Resendis-Antonio, 2013; Bordbar et al., 2014). Genome-scale models (GEMs) of metabolism (Rocha et al., 2008) provide a framework for the computation of the genotype-phenotype relationship in which various types of -omics data can be integrated along with organism-specific network reconstructions to generate tissue, cell, or organism specific *in silico* models (Feist et al., 2009; Chang et al., 2010; Agren et al., 2014; Fouladiha et al., 2015). These models can then be constrained by experimental measurements and computed in order to explore possible therapeutic applications, making use of the newest RNA sequencing and metabolomic data or *in vitro* experimentation. Such models will aid further understanding of MSCs metabolism under various external or internal conditions. Thus far, metabolic modeling has not been applied to the study of MSCs, but this area offers great possibilities for enhancing both research and therapeutic application of these cells.

In this review, we describe how the study of human MSC (hMSC) metabolism can be used to answer the fundamental question: “How can GEMs be used to optimize MSC therapeutics?” First, we describe the biology of MSCs, their differentiation and immunomodulation properties and their applications and limitations in regenerative medicine. Next, we detail how metabolism affects or can be used to manipulate these functions. We then discuss how mathematical modeling of hMSC metabolism can aid in developing pre-clinical and clinical experiments. Finally, we give our vision for the future of using metabolic modeling to study hMSCs and how the resulting insights could prove transformative for the field of regenerative medicine.

## Biology of Mesenchymal Stem Cells (MSCs)

**FIGURE 1:**
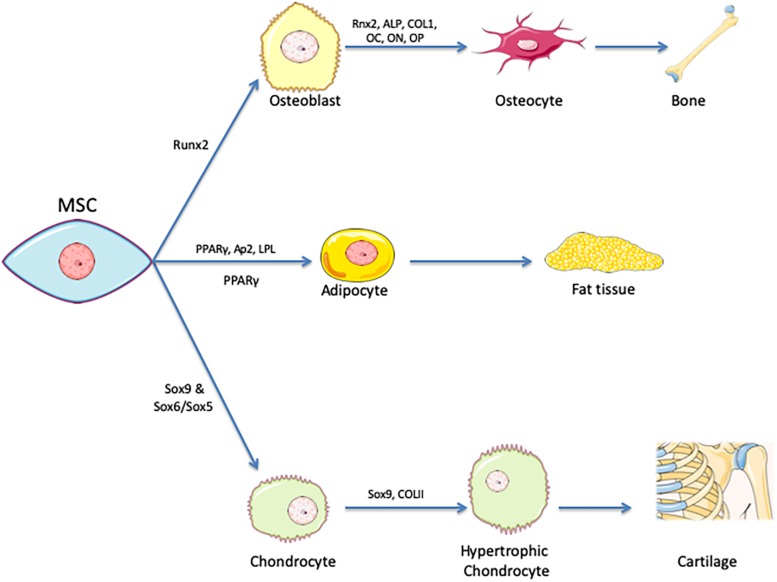
Tri-lineage encompasses differentiation of MSCs. Mesenchymal stem cells are identified by their ability to differentiate into chondrocytes, adipocytes, and osteoblasts that in turn develop into cartilage, fat tissue and bone. PPARγ is the master regulator of adipogenesis, Runx2 for osteogenesis and Sox9 for chondrogenesis. Various expression markers are used as indicators of successful differentiation.

Mesenchymal stromal cells comprise non-hematopoietic cells originating from the mesodermal germ layer and are capable of both self-renewal and multilineage differentiation into various tissues of mesodermal origin (Gazit et al., 2014). These multipotent cells can be isolated both from various adult tissues (e.g., skin, peripheral blood, bone marrow) and neonatal tissues (e.g., Wharton’s jelly, umbilical cord blood) (Nombela-Arrieta et al., 2011; Alberts et al., 2014). Despite the historical lack of consensus on methods for isolation, expansion, and characterization of hMSCs, the International Society for Cellular Therapy (ISCT) has produced minimal criteria to define hMSCs (Rosenbaum et al., 2008; Lin et al., 2013). The cells must be able to:

• Adhere to plastic and develop as fibroblast colony-forming units and differentiate into cells of mesodermal origin (i.e., osteocytes, chondrocytes, and adipocytes). See Figure 1.
• Express the surface markers CD73, CD90, and CD105 during *in vitro* culture expansion
• Lack expression of CD11b, CD14, CD34, CD45, CD19, and HLA-DR surface markers during *in vitro* culture expansion

It is likely that this definition will continue to evolve to account for new findings.

### Differentiation of MSCs

One of the identifying characteristics of MSCs is their ability to differentiate into cells of mesodermal origin (Nombela-Arrieta et al., 2011; Gazit et al., 2014). In addition to this hallmark trilineage differentiation, there have also been reports of differentiation toward other cell types of the ectodermal and endodermal origins, including tenocytes, cardiomyocytes, skeletal myocytes, smooth muscle cells, and neurons (Tatard et al., 2007; Galli et al., 2014; Ullah et al., 2015; Youngstrom et al., 2016). The actual functionality of the end product in this transdifferentiation is still debated.

Differentiation of MSCs is primarily induced through media supplementation (and, in some instances, mechanical stimulation), with different supplements being required for the various differentiations. Adipogenesis, for example, is induced through supplementation with dexamethasone, indomethacin, insulin, and isobutyl methyl xanthine. Osteogenic differentiation is induced by dexamethasone, ascorbic acid, β-glycerophosphate, and sometimes bone morphogenic protein 2 (BMP-2) (Ullah et al., 2015). The completion of differentiation is verified by checking the expression of characteristic cell type markers, such as lipoprotein lipase (LPL) for adipogenesis and alkaline phosphatase (ALP) activity for osteogenesis (Ullah et al., 2015). More detailed lists of differentiation-promoting components and the most characteristic markers used to measure level of differentiation are shown in Table 1.

**TABLE 1:** Summary of various inducing components, expression markers, and signaling pathways related to differentiation.

**Cell type resulting from differentiation**	**Differentiation-inducing components**	**Culturing time**	**Relevant expression markers**	**Most relevant reported signaling pathways and molecules**	**References**
Adipocytes	Dexamethasone Indomethacin Insulin Isobutylmethyl xanthine	14–21 days, with 2 phases (determination and terminal differentiation)	ap2 LPL PPARγ	β-catenin dependent Wnt (anti) Hedgehog (anti) NELL-1 (anti) BMP (pro)	James, 2013
Cardiomyocytes	5-azacytidine	28 days	α-MHC α-cardiac actin ANP cTnT Desmin	miR1-2 + Wnt/β-catening (pro) HDAC TGF-β VR-1 5-aza	Solchaga et al., 2011; Guo et al., 2018
Chondrocytes	Ascorbate 2-phosphate Dexamethasone Insulin Linoleic acid Selenious pyruvate Selenium TGF-βIII Transferrin	21 days, with 2 phases (pre – induction and terminal differentiation)	*Phase 1:* Collagen types I and II *Phase 2:* L-Sox5 Sox6 Sox9	*Phase 1 expression dependent upon:* TGF-β1,2 and 3 *Phase 2 expression dependent upon:* BMP2 IGF-I TGF-β1 Wnt/β-catenin (pro) PTHrp (anti)	Mackay et al., 1998; Antonitsis et al., 2008; Li and Dong, 2016
Hepatocytes	*Phase 1:* bFGF EGF Nicotinamide *Phase 2:* Dexamethasone Insulin Oncostatin M Selenium Transferrin	2 phases: differentiation (7 days) and maturation			Ullah et al., 2015
Neuronal cells	bFGF BME EGF FGF HGF Insulin LMX1A* NGF Retinoic acid Valproic acid				Ullah et al., 2015
Osteocytes	β-glycerophosphate Ascorbic acid BMP-2 Dexamethasone	21–35 days	ALP COL1 OC ON OP RUNX2	β-catenin dependent Wnt (pro) BMP (pro) Hedgehog (pro) NELL-1 (pro) TGF-β1 + Wnt/β-catening (anti)	Neve et al., 2011; James, 2013
Pancreocytes	Actavin A Nicotinaminde Sodium butyrate Taurine				Ullah et al., 2015
Skeletal/smooth muscle	NICD TGF-β				Ullah et al., 2015

*Note that this is not an exhaustive list.*

**FIGURE 2:**
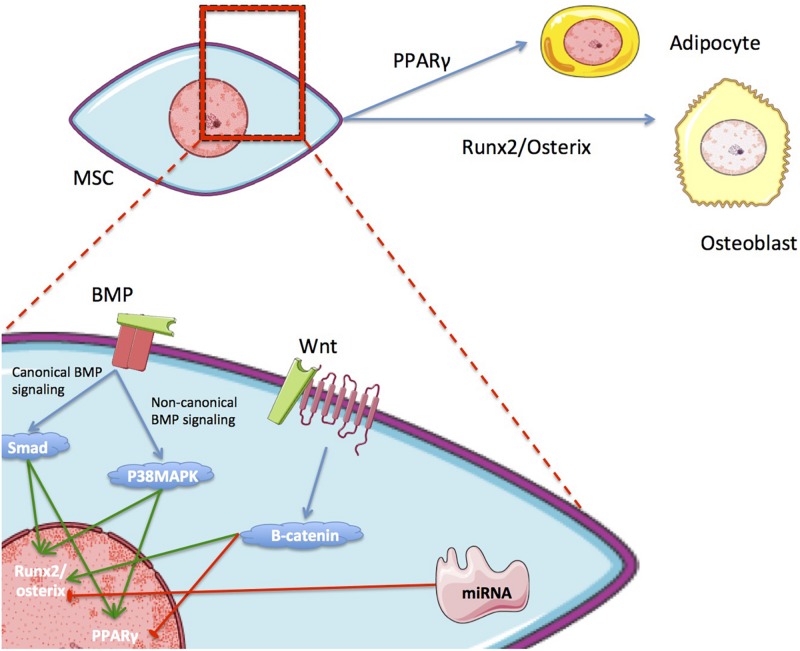
The inverse relationship of the main metabolic pathways in OD and AD. Differentiation toward one lineage can inhibit differentiation toward the other.

Differentiation is controlled by an interlinked set of regulatory molecules forming complex signaling pathways. These pathways are somewhat distinct between differentiation lineages, although there are important areas of overlap. This phenomenon is demonstrated by the inverse relationship that exists between pathways relating to adipogenic and osteogenic differentiation (Figure 2). Most differentiation pathways revolve around regulation of peroxisome proliferator-activated receptor (PPAR), which is the master regulator of adipogenesis, and runt-related transcription factor 2 (RUNX2), which is the master regulator of osteogenesis (Muruganandan et al., 2009; Neve et al., 2011; James, 2013; Hu et al., 2018). Further details on the most relevant reported signaling pathways and molecules for each type of differentiation are provided in Table 1.

### Immunomodulation of MSCs

**FIGURE 3:**
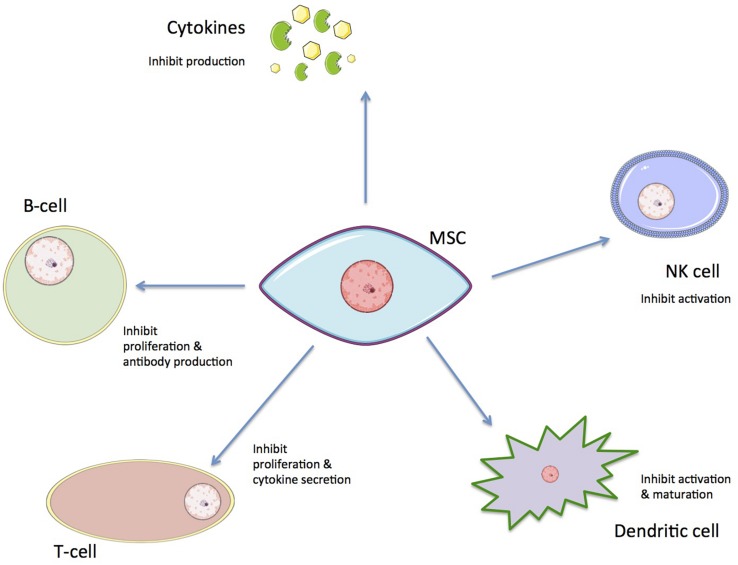
The immunoregulatory effects of hMSCs on immune cells.

Beyond their potential for differentiation, hMSCs have remarkable immunomodulatory properties; they possess the ability to inhibit or promote the immune response of the host’s body though mediated immunosuppression. These mechanisms include direct inhibitory effects and other indirect regulatory effects. This regulatory response involves inhibition of B and T cell proliferation, cytokine production inhibition, decreased natural killer (NK) cell activation, and dendritic cell maturation (Figure 3; Deng et al., 2005; Ma and Chan, 2016; Cunningham et al., 2018; Wang et al., 2018).

**FIGURE 4:**
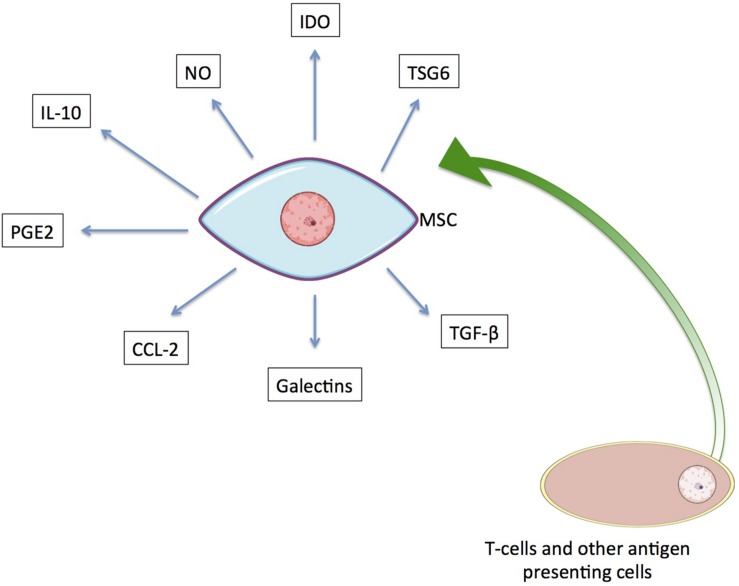
The immunoregulatory secretome of hMSCs. Known soluble factors secreted by MSCs upon activation by T cells and other antigen presenting cells are shown.

The immunomodulatory response of hMSCs is activated by inflammatory cytokines (e.g., IFN-γ, IL-1α, IL-1β, and TNF-α) that are secreted by T cells and other antigen-presenting cells (Ren et al., 2008; Németh et al., 2009). In response to their activation, MSCs secrete soluble immune factors capable of affecting both the innate and adaptive immune systems by mediating the subsequent regulatory responses of target cells (Figure 4; Kaundal et al., 2018; Wang et al., 2018). The immunoregulatory effects mediated in each instance are dependent on one or more of these secreted factors.

Indoleamine 2,3-dioxygenase (IDO) is one of the well-known paracrine factors released by hMSCs and has been shown to promote kidney allograft tolerance (Lan et al., 2010). It suppresses proliferation and activity of NK and T cells by its metabolic activity – converting tryptophan into kynurenine. In humans, IDO synthesis has been reported as a response of MSCs to pro-inflammatory cytokine production that suppresses the inflammatory response (Bernardo and Fibbe, 2013; Mbongue et al., 2015; Gao et al., 2016; Kaundal et al., 2018; Wang et al., 2018).

Another reported soluble factor with immunoregulatory effects is the unstable oxidative molecule nitric oxide (NO), which is generated by NO synthase. Inducible NO synthase (iNOS) is responsible for the immunomodulatory effect of NO. Increased secretion of NO results in modulation of both the proliferation and function of T cells. At very high concentrations, it can lead to the apoptosis of immune cells (Bernardo and Fibbe, 2013; Gao et al., 2016; Kaundal et al., 2018; Wang et al., 2018). A list of the known soluble paracrine factors secreted by hMSCs that are involved in immunoregulation is provided in Table 2, along with their related effects.

**TABLE 2:** A list of inflammatory cytokines that activate immunoregulatory state of hMSCs, major known soluble paracrine factors secreted by hMSCs, and a summary of their biological functions.

**Immunosuppressive factors secreted by MSCs**	**Summary of biological function**	**Activating inflammatory cytokines**
CCL2	Promotion of monocyte migration. Suppression of activation and mitigation of TH17 cells.	IFN-γ, IL-1α, IL-1β, TNF-α
Galectins	Suppression of the immunomodulatory effects of T cells.	
IDO	Suppression of the effects and proliferation of immune cells.	
IL-10	Suppression of immune cell apoptosis.	
NO	Promotion of immune cell apoptosis. Suppression of proliferation and modulation of T cells.	
PGE2	Suppression of NK cell cytolytic activity and T cell proliferation.	
TSG6	Overall anti-inflammatory effect.	
TGF-β	Inhibition of mast cell degranulation, NK cell activation and proliferation, and Treg induction.	

### Homing Effects of MSCs

Mesenchymal stromal cells secrete paracrine factors that promote tissue repair. In response to physical tissue damage, MSCs secrete factors that allow them to navigate to the site of injury, referred to as homing (Ullah et al., 2015). An example of a homing molecules used by MSCs are the chemokine receptors CXCR4 and CXCR7, which both bind to stromal cell-derived factor (SDF-1) on endothelial cells; this is a critical step in facilitating homing of MSCs to various tissues (Ullah et al., 2019). Homing is generally considered to be beneficial for tissue repair (Ullah et al., 2015) due to the interaction of the cells with the host tissue via secretion of trophic and paracrine factors (Ullah et al., 2015; Moreira et al., 2017). Engrafting or migration of hMSCs in experimental settings relies in part on this phenomenon in combination with the direct delivery of cells.

The homing effect and subsequent migration of hMSCs has been observed. However, the mechanisms behind it are not well understood. Only a small percentage of systemically administered cells manage to reach target tissue and remain there (De Becker and Riet, 2016; Moreira et al., 2017). For the most part, this low success rate has been ascribed to low expression levels of homing molecules, loss of expression of homing molecules during *in vitro* expansion, and cultural heterogeneity of the hMSCs. Cells derived from different sources seem to express different profiles of the homing molecules (De Becker and Riet, 2016; Moreira et al., 2017).

### MSCs as a Novel Tool in Regenerative Medicine

Regenerative medicine is considered a novel frontier in medical research (Maienschein, 2011; Sampogna et al., 2015). It combines the knowledge and application of various fields such as tissue engineering, cell transplantation, stem cell biology, biomechanics, prosthetics, nanotechnology, and biochemistry to replace or restore human cells, tissues, or organs to their normal functions (Sampogna et al., 2015). A variety of regenerative medicine therapies are available (see Lonner et al., 2000; Blais et al., 2013; Zhang X. et al., 2013; Trushina and Mielke, 2014; Björnson et al., 2016; Moreira et al., 2017), but their success has been limited by functional obstacles that increase the risk of harm to patients and reduce their efficacy as a therapeutic (Neman et al., 2012; Campana et al., 2014; Goldberg et al., 2017; Cunningham et al., 2018; Solarte et al., 2018). Despite recent progress, there is obvious room for improvements regarding both the safety and efficacy of therapies for patients. The multipotency, high proliferation potential, paracrine effect, and immunomodulatory activity of hMSCs (Rosenbaum et al., 2008; Ullah et al., 2015; Fitzsimmons et al., 2018) have led to development of MSCs as a tool for use in regenerative medicine. Thus, MSCs are considered ideal candidates for immunotherapy and tissue engineering.

Recent advancements have allowed researchers to overcome initial obstacles in the use of MSCs. Numerous clinical trials have assessed their safety and found that transfusions using these cells are safe (Neman et al., 2012; Zhao et al., 2016). Various studies have developed isolation and culture approaches along with various possible mechanisms of delivery. These studies have shown that long-term culture of MSCs is possible without losing the cells functional, phenotypical, or morphological features (Bernardo et al., 2007).

Further, MSCs are becoming readily available for biomedical research. There is growing interest in the use of placental- and umbilical cord-derived hMSCs due to the relatively high availability of discarded tissue associated with births (Moreira et al., 2017); however, the variance in phenotypic properties (if any) between hMSCs derived from different sources is an important open question. Bone marrow- (BM-) and adipose tissue-derived (Ad-) hMSCs are the most favored stem cell types in both tissue engineering and cell-based medicine for a variety of reasons, despite the invasive procedures required for tissue collection (Fitzsimmons et al., 2018): (1) the total cell number that can be harvested each time is higher than with other stem cells; (2) the frequency of cells of interest is higher than with other stem cells; and (3) Ad-hMSC harvesting can be performed as part of some elective cosmetic surgeries (e.g., liposuction) (Fitzsimmons et al., 2018). Ad-hMSCs have been shown to have increased capacity for adipogenic differentiation *in vitro*, while BM-hMSCs have increased capacity for osteogenic and chondrogenic differentiation (Liu et al., 2007). Through a comparative study on the immunomodulatory abilities of cells derived from the same donor bit different tissues, Valencia et al. (2016) determined that Ad-hMSCs have a higher capacity for inhibiting dendritic cell differentiation than do BM-hMSCs, while BM-hMSCs displayed a higher capacity for inhibition of NK cell cytotoxic activity; these results have been corroborated by several independent groups (Ivanova-Todorova et al., 2009; Blanco et al., 2016). These observations—whether relating to proliferation potential or direct therapeutic application abilities—highlight the importance of choosing the optimal cell source for a particular clinical circumstance.

#### Efficacy of Cell Engraftment vs. Paracrine Factors

**FIGURE 5:**
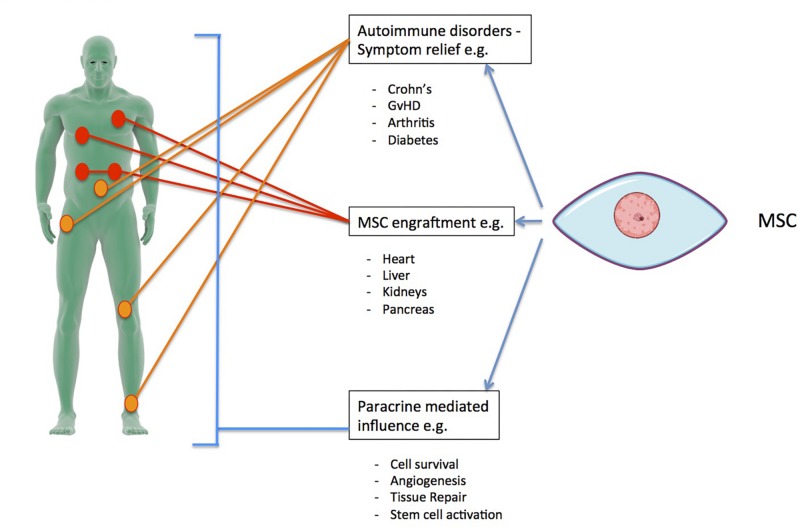
Examples of therapeutic applications for hMSCs, including organs where cell transplants have been used for engraftment, some autoimmune disorders where the immunoregulatory and differentiation abilities of hMSCs have been utilized, and some of the paracrine factor-mediated influences that can be used in a therapeutic setting.

For the last several decades, the therapeutic potential of hMSCs has been focused on cell transplantation, adding hMSCs to a recipient donor site for repair via regeneration, differentiation, and immunomodulation (Lukomska et al., 2019). Co-culturing in animal studies has shown that hMSCs can induce tissue regeneration to some extent in the heart (Rose et al., 2008), kidneys (Qian et al., 2008), and liver (Cho et al., 2009) through infiltration and replacement in damaged or injured tissue by multipotent hMSCs (see Figure 5). Increasing attention, however, has lately been given to the immunomodulatory and suppressive capabilities of hMSCs, especially with regards to their paracrine factors (Németh et al., 2009; Cunningham et al., 2018; Kaundal et al., 2018). Currently, approximately 10% of the clinical trials registered in the United States are using MSCs to study immunological disease. Through their ability to decrease inflammation and general inhibitory functions, hMSCs have been utilized as contributing factors for various immune disorders for symptom relief. Such disorders include type 1 and type 2 diabetes (Moreira et al., 2017), acute graft versus host disease (GvHD) (Gao et al., 2016), arthritis (Burke et al., 2016), allograft rejection (Munir and McGettrick, 2015), and Crohn’s disease (Ibraheim et al., 2018). The possible immunomodulatory effects of hMSCs have been found to be dependent upon the source of the hMSCs as well as their immediate microenvironment (Bortolotti et al., 2015). The microenvironment is dependent upon the individual inflammatory profile of the host, which is potentially related to any disease pathogenesis present. This variability leads to varied cytokine profiles that are, at least in part, responsible for the difficulties of using MSC therapy effectively in both preclinical and clinical situations (Kaundal et al., 2018). Some of these challenges may be overcome by personalizing each case (i.e., tailoring each therapy to the inflammatory environment of the recipient patient).

There has been a recent paradigm shift away from the primary aim of hMSC transplantation being tissue repair by engraftment toward the use of hMSCs to promote healing via their secretion of paracrine factors. In many therapeutic contexts, it is now recognized that MSCs exert their healing effects through paracrine signaling and cell-to-cell contact, not by replacing cells (Fitzsimmons et al., 2018). There are a few notable examples using hSMCs as paracrine-mediated treatment currently in development (Amorin et al., 2014; Hofer and Tuan, 2016; Archambault et al., 2017; Moreira et al., 2017; Cunningham et al., 2018; Ibraheim et al., 2018; Solarte et al., 2018). The reported success of these studies indicates that the MSC secretome exerts beneficial effects that may be exploited by therapeutic applications.

### Existing Challenges and Problems

Despite the initial successes demonstrated in animal and early clinical trials regarding the safety and efficacy of hMSCs, a number of challenges, problems, and unanswered questions remain. In order for *in vitro* cultured MSCs to engraft after implantation or to secrete their beneficial factors, they must be able to survive. The transplantation procedure itself exerts various direct mechanical and chemical stresses on the cells, and the treated tissue offers a comparatively much harsher environment than the standard culture surroundings that cells experience *in vitro*. In tissues *in vivo*, there are various negatively impacting stressors, such as hypoxia, inflammation, decreased energy/nutritional availability, and high acidity. Various strategies to enhance survival and overcome these adverse conditions have been developed, including preconditioning, genetic modification, and supportive biomaterials as a delivery device (Baldari et al., 2017; Moreira et al., 2017).

It is possible that donor variability and differences in isolation site have an effect on experimental outcomes, though the extent of these effects is not yet well understood. This uncertainty may be exacerbated by heterogeneity in the origins of MSCs used in tests (Bortolotti et al., 2015; Lukomska et al., 2019). Further, there is a lack of knowledge of the optimal dose and frequency required for hMSC transplantation (Lee, 2018; Lukomska et al., 2019). In addition, generating high doses of hMSCs requires cellular expansion on a large scale. Despite being proven to retain their characteristics over long term expansion (Bernardo et al., 2007), MSCs eventually become senescent (Turinetto et al., 2016). Senescent cells have undergone functional changes. Firstly, differentiation potential usually decreases due to accumulation of oxidative stress and dysregulation of key differentiation regulatory factors. Secondly, both migratory- and homing-related abilities of hMSCs are reduced as they move into senescence. Finally, the secretome of hMSCs becomes compromised in senescence. Many of the factors that are present in the senescent MSC secretome can exacerbate an inflammatory response at a systemic level and so promote either migration or proliferation of cancer cells (Turinetto et al., 2016).

In many cases of MSC transplantation, there is little to no integration of the transplanted cells, and cells that are retained are observed to have a short survival time in some cases (Li et al., 2016; Lukomska et al., 2019). Even though more than two thousand patients have received either autologous or culture expanded allogeneic MSCs, long-lasting observations are lacking in many cases (Lukomska et al., 2019). This lack of data indicates that more progress is difficult. Tumor support due to the immunosuppressive effects of MSCs and the related possibility of tumorigenicity of such a therapy has been reported (Barkholt et al., 2013). There have also been reports of BM-hMSC-induced liver fibrosis (Russo et al., 2006). These issues must be addressed by long-term studies regarding safety of use.

While the use of hMSCs holds great promise in regenerative medicine, the hurdles and unanswered questions outlined in this section still linger. perhaps the greatest barrier preventing widespread and successful implementation of hMSCs as tools to enhance and develop therapeutics is the critical gap in knowledge of hMSC metabolism. During differentiation, we can observe a metabolic shift in hMSCs from using only glycolysis to a mix of glycolysis, oxidative phosphorylation, and beta fatty acid oxidation to produce energy. However, in order to have immunomodulatory effects (via paracrine factors) on a host‘s immune system, hMSCs must be mainly in a glycolytic state. Further, it is not well understood to what extent energy metabolism is mixed at different stages of differentiation (early versus late) or whether it is dependant upon the type of differentiation the cells are undergoing. An understanding of how cell source and age affects differentiation and if different states affect the survival and function and, therefore, usability of cells *in vivo*. In the following section, we delve into some of these gaps and discuss how acquiring a deep understanding of underlying mechanisms can help unlock the therapeutic potential of hMSCs in regenerative medicine.

## Metabolism of MSCs

### MSC Function Is Linked to Metabolism

The proliferation, differentiation, and immunomodulatory functions of hMSCs are linked to cellular metabolism (dos Santos et al., 2010; Estrada et al., 2012; Beegle et al., 2015). Emerging evidence suggests that hMSCs are metabolically heterogeneous and that these differing metabolic states impact both differentiation ability and capacity for immunomodulation (Agathocleous and Harris, 2013; Liu et al., 2019). To date, studies have focused primarily on BM-hMSCs and Ad-hMSCs.

There is strong evidence suggesting that, in their undifferentiated state (while undergoing proliferation), hMSCs rely primarily on glycolysis for energy production. This phenotype has been demonstrated in BM-hMSCs, which show a preference for glycolysis during proliferation (Pattappa et al., 2011; Buravkova et al., 2013; Shum et al., 2016; Zhu and Thompson, 2019) but shift to a more oxidative phosphorylation (OxPhos)-dependent metabolism during osteogenic and adipogenic differentiation (OD, AD) (Meleshina et al., 2016; Shum et al., 2016). Similar findings have been reported for Ad-hMSCs (Meleshina et al., 2016; Meyer et al., 2018). Proliferating Ad-hMSCs were found to have a preference for glycolysis even under aerobic conditions, while during OD the cells increased both glycolysis and mitochondrial metabolism, including the processes of OxPhos and fatty acid β-oxidation. However, when Ad-hMSCs underwent AD, they showed a decreased capacity for the pentose phosphate pathway (PPP) and glycolysis, while mitochondrial enzyme activities increased, indicating an increased capacity for oxidative phosphorylation and β-oxidation (Meleshina et al., 2016; Meyer et al., 2018).

The ability of BM-hMSCs to differentiate has also been shown to be affected by mitochondrial functions (Zhang Y. et al., 2013; Li et al., 2017). Consistent with reports that proliferating hMSCs have a glycolytic phenotype, undifferentiated cells have high levels of hypoxia-inducible factor 1 (HIF-1), a transcriptional regulator central to regulation of genes that are involved in hypoxic responses. It is also a crucial physiological regulator of anaerobic metabolism (Gaspar and Velloso, 2018). Cells undergoing OD downregulate HIF-1. Downregulation of HIF-1 seems to be required for the activation of mitochondrial OxPhos, an oxygen-dependent pathway (Shum et al., 2016).

The mitochondria of hMSCs seem to be primarily inactive while cells remain in their proliferation stage, during which metabolic pathways related to glycolysis and its associated signaling pathways required for adenosine triphosphate (ATP) generation and general anabolitic activity are most active (see Table 3). Glycolytic metabolism also seems to be a requirement for hMSCs to be able to sustain immunosuppressive factor secretion (Liu et al., 2019). Secretion of immunomodulatory factors is only possible when hMSCs have been activated, such as by IFN-γ (Waterman et al., 2010). Liu et al. (2019) utilized IFN-γ treatment to cause immune polarization in hMSCs leading to remodeling of metabolic pathways toward glycolysis (reducing TCA cycle metabolism), a requirement for sustained immunosuppressive factor secretion. The activated cells were measured to have increased lactate levels, glucose consumption, and acidification rate. Increased expression of glucose transporter 1 and hexokinase isoform 2 (key enzymes in glycolysis), along with reduced electron transport and OxPhos, was also observed. These are all indicators of increased glycolytic activity (Liu et al., 2019). MSCs with a glycolytic phenotype are also able to sustain IDO production and the exposure to IFN-γ inhibited activity of the mitochondrial electron transport chain (complexes I or III), blocking OxPhos and reducing mitochondria-related reactive oxygen species (mROS). This reduces the effects of mROS that are key to metabolic remodeling in differentiation. Liu et al. (2019) further showed that Akt/mTOR signaling pathway activation is required to induce metabolic reconfiguration, specifically IDO and Prostaglandin E2 (PGE2) production. PGE2 increases in response to increased aerobic glycolysis. The immune response of hMSCs treated with IFN-γ is altered if the metabolic reconfiguration induced by Akt/mTOR is disrupted.

**TABLE 3:** List of common signals in metabolism and the major metabolic pathways effected.

**Signal**	**Metabolic pathways regulated by the signal**
AMPK	Inhibition of glycolysis and fatty acid synthesis. Promotion of fatty acid oxidation.
Hedgehog	Stimulation of glycolysis.
HIF	Redirection of energy metabolism from OXPhos to glycolysis.
mTOR	Stimulation of glycolysis, lipid synthesis, protein synthesis, and pyrimidine synthesis.
Myc	Stimulation of glycolysis, glutaminolysis, and nucleotide synthesis.
PI3K	Stimulation of glucose uptake, fatty acid synthesis, and glycolysis.
Ras	Stimulation of glucose uptake and PPP. Regulation of glutaminolysis.
Sirtuins	Regulation of TCA cycle, glycolysis, and fatty acid oxidation.

The effect of interferon regulation on hMSC metabolism can be varied. IFN-γ has also been used to inhibit proliferation and alter AD, OD, and neural differentiation (ND) by activating IDO (Croitoru-Lamoury et al., 2011). The kynurenine pathway (KP), along with IDO1 and IDO2, is expressed in hMSCs and highly regulated by both IFN-γ and IFN-β. IFN-γ licensing of hMSCs results in inhibited proliferation via activation of the KP and subsequently IDO, and inhibits the cell potential for OD and AD. In contrast to IFN-γ licensing, IFN-β treatment managed to increase expression of adipogenic markers (Croitoru-Lamoury et al., 2011).

IFN-β has been shown to enhance immunomodulatory functions of hMSCs in other reports. Vigo et al. (2019) demonstrated IFN-γ induced expression of secretory leukocyte protease inhibitor (SLPI) and hepatocyte growth factor (HGF), soluble mediators that are involved in both immune and regenerative functions of hMSCs. Simultaneously, IFN-β induced the activity of mTOR, increasing the glycolytic capacity of the cells. This energy metabolic modification improved the cells’ ability to control T cell proliferation, yet another indication of a link between high glycolytic capacity and immunomodulatory capabilities (Vigo et al., 2019).

Overall reports discussing hMSC metabolism seem to, for the most part, agree that during proliferation the cells primarily generate ATP through glycolysis. However, upon initiation of differentiation, cells seem to turn toward mitochondrial metabolism, with reported increases in metabolism and biogenesis indicating the importance of mitochondrial activity when it comes to hMSC functionality (Shum et al., 2016).

There is not much known about whether amino acid metabolism is affected during functional progression of hMSCs or what effects they induce if modified through metabolic changes. For example, El Refaey et al. (2015) studied the aromatic amino acids tryptophan and tyrosine, finding that oxidation (via cell senescence) disrupted their anabolic effects on BM-MSCs. By using mouse BM-MSCs, they were able to examine effects of oxidized dityrosine and kynurenine of proliferation and differentiation and found that these oxides inhibited BM-MSC proliferation, ALP expression and activity and expression of osteogenic markers. Yue et al. (2018) studied fatty acid related gene expression and compositions of fatty acids during adipogenesis of bovine Ad-MSCs and found that lipid-related gene expression and fatty acid composition changed noticeably during the early stages of differentiation (e.g., there was increased expression of *de novo* lipogenesis-related genes, and thus *de novo* lipogenesis produced fatty acid elongation and desaturation) before returning to normal (e.g., proportions of saturated fatty acids, monounsaturated fatty acids, and polyunsaturated fatty acids returned to initial levels in later stages). Their conclusion was that *de novo* lipogenesis and desaturation comprised the major fatty acid flux during adipogenic differentiation of bovine Ad-MSCs.

Ornithine decarboxylase (ODC) and polyamine biosynthesis are important in the proliferation of stem cells (Tsai et al., 2015). The role of ODC regarding differentiation has not been fully explored but is considered to be diverse. Through the study of inhibition of ODC’s irreversible inhibitor, α-difluoromethylornithine, Tsai et al. (2015) hypothesized that inhibition of ODC and the accompanying depletion of exogenous polyamines might be correlated with the osteogenic induction of BM-hMSCs, and demonstrated (in BM-hMSCs) that decreases in the expression of PPAR- γ and ODC along with an accompanying reduction in polyamines, are responsible for enhanced osteogenesis.

### MSC Functionality Is Greatly Impacted by Mitochondrial Activity

As suggested in section “MSC Function Is Linked to Metabolism,” active mitochondria are necessary for successful differentiation. Accumulating evidence indicates that mitochondrial enzymes and regulatory pathways are of great importance for MSCs in proliferative and differentiating states (Chen et al., 2008; Buravkova et al., 2013; Li et al., 2017). Mitochondria have been found to be crucial for sufficient ATP production to support OD, in addition to other mechanisms. Active mitochondria support OD by promoting β-catenin acetylation and, therefore, its activity. β-catenin is an important signaling pathway in osteogenesis (Shares et al., 2018). In osteogenesis, a mechanism of OD induction is to induce mitochondrial OxPhos by replacing glucose with galactose. This switch also stimulates β-catenin signaling and β-catenin acetylation. Increased β-catenin acetylation is the mechanism of osteogenesis driven by mitochondrial OxPhos (Shares et al., 2018). This acetylation increases during osteogenesis (BM-hMSCs). Active mitochondria may also support other osteogenic pathways by providing acetyl groups.

Other enzymatic activity has been confirmed that further supports the importance of mitochondrial activation for MSC functionality. Creatine kinase (CK) activity, which is involved in buffering and recovery of ATP, has been reported in Ad-hMSCs. It stimulates glycogenolysis by increasing cytoplasmic concentration of inorganic phosphate. Activity of CK was found in both differentiated and proliferating Ad-hMSCs, with more mitochondrial CK activity in AD cells. This further supports the theory of a shift toward oxidative metabolism/mitochondrial metabolism during differentiation of MSCs (Meyer et al., 2018).

Through the reversible mitochondrial nicotinamide adenine dinucleotide phosphate (NADP)-dependent reaction of isocitrate dehydrogenase (NADP-IDH), an anaplerotic pathway exists that forms isocitrate from glutamine through a process called glutaminolysis. Through this pathway, glutamine can compensate for the lack of glucose for both ATP production and anabolic precursor supply (Smolková and Ježek, 2012). This pathway is active in MSCs during OD, indicating yet another important role that mitochondria play when it comes to provision of sufficient ATP to ensure successful differentiation.

Reactive oxygen species (ROS) are known to serve as signaling molecules capable of regulating biochemical pathways that are a part of normal cell function. They are particularly important in metabolism and inflammatory signaling (Forrester et al., 2018). Regulation of mROS levels also contributes to determination of differentiation outcome. For a long time, these molecules were considered to be harmful to cells, inducing organismal death and dysfunction, but more recent reports suggest that excess mROS impair OD and promote AD by inhibiting Hedgehog signaling (a pathway essential for bone development and maintenance) (Li et al., 2017).

### Possible Ways to Achieve Metabolic Manipulation

**FIGURE 6:**
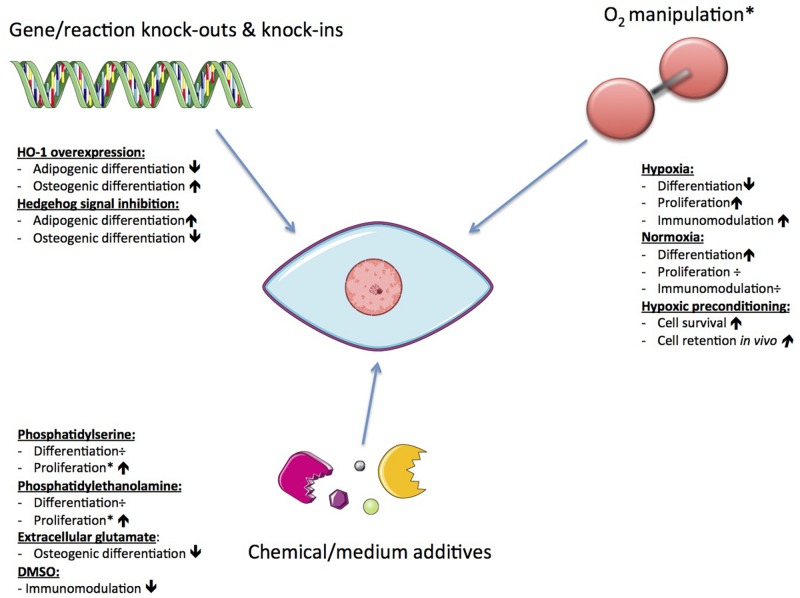
Possible ways to achieve metabolic manipulation of hMSCs. The resulting effects may vary depending on the original cell source.

Since functionality and survival of hMSCs is affected by changes in their metabolism, there is the potential to enhance the efficacy of hMSC therapies through manipulation of metabolism. hMSCs can effectively reconfigure metabolism to respond to the biochemical demands of tissue repair, be it secretion of immunomodulatory factors or integration and differentiation toward tissue specific cell types (Mylotte et al., 2008; Zhu et al., 2014; Yuan et al., 2019). Currently, the most extensively studied subtypes are BM-hMSCs and Ad-hMSCs, but even these subtypes have not been exhaustively studied. Evidence indicating the importance of both the enzymes and mitochondrial pathways support its significance for proliferation and differentiation of hMSCs (Chen et al., 2008; Buravkova et al., 2013; Li et al., 2017). In addition, several critical MSC functions are not only influenced by internal cellular mechanisms, but also by external ones (mechanical and biochemical) such as the composition of its microenvironment (Bloom and Zaman, 2014). Previous work has explored various ways of affecting the mechanisms controlling MSC metabolic function (see Figure 6).

One such approach is to alter the functional capacity of MSCs through oxygen manipulation, due to the importance of mitochondria and mROS discussed above. Muñoz et al. (2014) investigated the effect of oxygen levels on metabolic phenotypes of hMSCs. Oxygen is a ubiquitous regulator of cellular metabolic activity and of the survival, function, and differentiation of hMSCs. Using both normoxic and hypoxic conditions, they found contrasting metabolic profiles for hMSCs during proliferation versus OD. The key difference was found in the coupling of glycolysis to the TCA cycle, glutaminolysis, and malate-aspartate shuttle. In response to low oxygen levels, undifferentiated hMSCs showed increased consumption of both glucose and glutamine that activated the malate-aspartate shuttle in order to accommodate increased cytosolic production of nicotinamide adenine dinucleotide + hydrogen (NADH) and transport glutamate and reducing equivalents into the mitochondrial matrix for oxidation (Muñoz et al., 2014). Low oxygen also activates HIF-1, reducing pyruvate dehydrogenase activity so that transport of glucose derived carbons into the TCA cycle decreases (Estrada et al., 2012). These metabolic characteristics allow increased proliferation under hypoxic circumstances, allowing cells to survive in an ischemic environment.

Similar findings were reported for proliferation of cells grown under hypoxia (dos Santos et al., 2010). For cells in OD, hypoxia induces a more significant block of carbon flow from glycolysis into the TCA cycle, compared to undifferentiated cells. This is demonstrated by a greater rise in lactate levels. The carbon flow blockage results in lower citrate levels and less production of reduced cofactors [e.g., NADH and flavin adenine dinucleotide (FADH_2_)] involved in OxPhos. The lower citrate levels indicate a more pronounced metabolic uncoupling of glycolysis and TCA cycle for cells in OD compared to undifferentiated cells (Muñoz et al., 2014). This observation of a tight coupling of glycolysis and TCA cycle in cells undergoing OD compared to proliferating hMSCs suggests a stronger dependence on oxygen during OD (Muñoz et al., 2014). This dependency has been shown independently (Buravkova et al., 2013). Permanent oxygen deprivation resulted in the attenuation of cellular ATP levels, leading to diminished mitochondrial ATP production and stimulation of glycolytic ATP production. The attenuated cellular ATP levels stimulated a proliferation state of the hMSCs and reduced the differentiation capacity, indicating that low ATP levels (arising from glycolysis only) are sufficient to maintain the cells’ uncommitted state (Buravkova et al., 2013). Hypoxia has also been used to precondition MSCs to enhance their survival and cell retention *in vivo* via induction of metabolic changes (Beegle et al., 2015).

Reactive oxygen species are known to play a role in the mediation of both pathophysiological and physiological signal transduction (Forrester et al., 2018). The subcompartments in cells (e.g., peroxisome and mitochondria) that produce ROS are often associated with metabolism. Mitochondria-related ROS are able to influence metabolic processes on their own, and so have an effect on differentiation and immunomodulation of hMSCs. Studies have shown that by using mitochondrial-targeted antioxidants, AD may be inhibited; however, as mentioned in section “MSC Functionality Is Greatly Impacted by Mitochondrial Activity,” excess mROS impair OD and promote AD by inhibiting Hedgehog signaling (Li et al., 2017). The role of mROS in chondrogenic differentiation (CD) is less well known (Li et al., 2017). Takarada-Iemata et al. (2011) found that through sustained exposure to glutamate, a significant decrease in osteoblastic marker expression could be induced. This happened in association with a reduction of intracellular glutathione (GSH) levels, but without affecting adipogenic marker expression. This finding suggests that extracellular glutamate preferentially suppresses osteoblastogenesis over adipogenesis in MSCs through the cysteine/glutamate antiporter (Takarada-Iemata et al., 2011).

The effects of other chemical stimuli have also been reported. In contrast to the inhibiting effects of extracellular glutamate on OD, it was reported that by inducing overexpression of heme oxygenase-1 (HO-1) OD of BM-hMSCs may be enhanced and adipogenesis decreased (although no mechanism was determined) (Barbagallo et al., 2010). HO-1 is a nuclear factor erythroid 2-related factor 2 (Nrf2)-regulated gene that plays a critical role in preventing vascular inflammation. It also has important antioxidant, anti-inflammatory, antiproliferative, and antiapoptotic effects in vascular cells (Araujo et al., 2012). Recent reports suggest that frozen or cryopreserved hMSCs are therapeutically less effective than freshly harvested MSCs (François et al., 2012). It seems that dimethyl sulfoxide (DMSO), a commonly used cryopreservative solution, decreases metabolic and immunosuppressive properties of hMSCs, while valproic acid (VPA) pre-treatment enhances both (François et al., 2012). Moreover, the T cell suppressive capacity of hMSCs *in vivo* is related to the cells’ glycolytic and respiratory capacity, in contrast to their IDO dependence *in vitro*. This observation, therefore, leads to speculation that hMSCs may only be able to induce immunoregulatory effects when undifferentiated.

Metabolism in MSCs is a complex and dynamic system. We have outlined several gaps in the collective knowledge of MSC metabolism that are actively being addressed by the community. As we gain insights into questions regarding the primary energy-generating pathway(s) utilized during differentiation, we will move closer to manipulating these systems. However, we will need a holistic perspective that integrates knowledge at the various biological levels of MSC differentiation.

## Mathematical Modeling of Human Metabolism

A bottom-up systems biology approach allows for a mechanistic understanding of a system (Westerhoff and Palsson, 2004). Such mathematical models can predict potential interventions, potentially providing insights into how to successfully manipulate MSCs for therapeutic applications. Over the last few decades, many individual components of MSC biology have been studied in detail. However, to predict a cell’s phenotype, it is necessary to understand all of the systemic interactions of environmental and cellular components that contribute to that phenotype (Bordbar et al., 2014). A combination of high-throughput -omics technologies, enabling the collection of large data sets, and improved computational modeling methods to holistically analyze that data have made systems biology possible (Henry et al., 2010; Resendis-Antonio, 2013).

**FIGURE 7:**
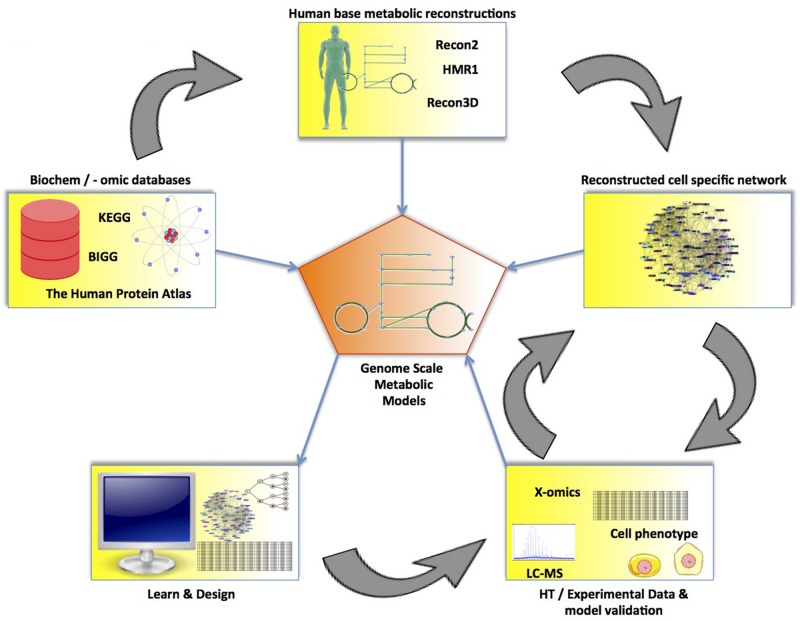
The process of reconstructing a genome-scale metabolic model (GEM). Using a base human metabolic reconstruction along with experimental data and biochemical databases (e.g., KEGG or BiGG), a constraint-based model can be created. From this model, a GEM is derived, from which cellular functions can be studied. Newly acquired knowledge from the GEM can be used to create cell experiments to further validate or improve the model. The validated GEMs can then be used to further study cells for use in regenerative medicine.

**FIGURE 8:**
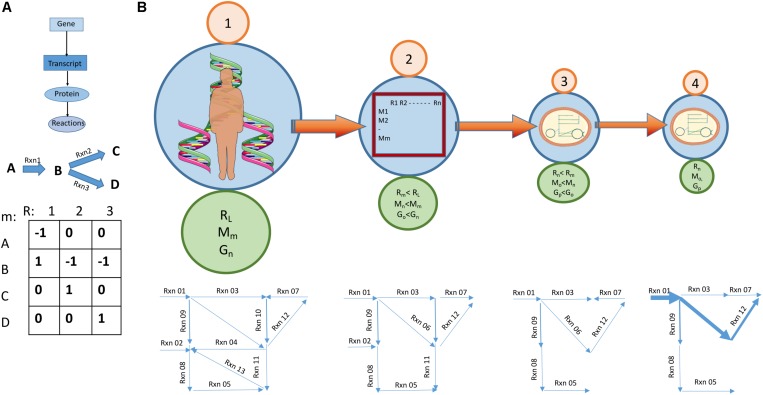
Going from a large base model to a cell specific GEM. **(A)** Genome scale metabolic models build on the gene-protein reaction association and represent a set of reactions as a matrix with linked genes. Synthesis of metabolites is designated by a positive number and breakdown with a negative number. **(B)** Going from a large base model to a cell/condition specific model. (1) A relevant base model is chosen. This model is a summary of known metabolic reactions and forms a species specific metabolic reconstruction. This base model usually has the highest count of genes, reactions and metabolites. (2) A process aiming at reducing the size of the model starts. By considering biochemical and biophysical constraints, e.g., thermodynamic feasibility, some reactions stop being reversable and the reconstruction becomes a model. Other constraints applied at this stage relate to stoiciometricity and enzyme capacity. The number of genes, reactions and metabolites is reduced. (3) Data about transcripts and proteins present in a cell type and the availability of nutrients in medium lead to the removal of yet more reactions. A cell specific model is created. (4) Metabolomic data specific to the condition the cell is supposed to represent allows the magnitude of reactions to be predicted. Some will have higher flux rates than others. More reactions may be removed at this stage. The model now becomes condition specific.

The first step in modeling metabolism at the genome-scale is to reverse engineer the network structure (Reed and Palsson, 2003; Resendis-Antonio, 2013; Bordbar et al., 2014; Yurkovich and Palsson, 2016). This reconstruction process starts with collecting all annotated components of the genome and experimental evidence of biochemical reactions for the organism of interest (Thiele and Palsson, 2010). Further constraints are placed on the network based on biochemical knowledge—including stoichiometric constraints (e.g., mass and charge balance of reactions), thermodynamic constraints, and enzymatic capacity constraints (Reed and Palsson, 2003; Rocha et al., 2008; Oberhardt et al., 2009; Thiele and Palsson, 2010)—eventually resulting in a genome-scale model (GEM) of metabolism (see Figure 7). Transcriptomic and proteomic data is then used to select which of these reactions are active in a given phenotype, based on the presence of the enzyme that catalyzes the reaction. Metabolomic data may be used to constrain which metabolites should be produced or consumed by the cell being modeled (Bordbar et al., 2017). The resulting GEM can then be used with a variety of computational approaches, such as flux balance analysis (FBA), to determine the flux state (i.e., pathway usage) of the entire metabolic network (see Figure 8).

This ability to integrate information from multiple types of -omic data with previously acquired detailed biochemical data makes metabolic modeling a powerful technique to answer biological questions regarding how phenotypes occur due to genetic mutation or environmental perturbations (Thiele and Palsson, 2010). As further data is obtained, the model will more closely represent the intended physiological conditions. An updated model may produce novel hypotheses which can suggest new experimental directions. Establishing this feedback between experimental design and computational evaluation is valuable and enables a better understanding of how cells—including MSCs—organize their metabolic system in response to shifts in environment and functional demands (Reed and Palsson, 2003; Resendis-Antonio, 2013). Furthermore, the ability to contextualize models based on information at various levels from genomic to environmental has the potential to allow models to inform personalization of medicine, for example by predicting potential genetic markers of a successful MSC donor.

### Existing Human Models

#### Genome Annotation Efforts Led to the First Human Metabolic Reconstructions

Various community-driven efforts have led to several reconstructions of the global human metabolic network (Romero et al., 2004; Duarte et al., 2007; Ma et al., 2007; Agren et al., 2012; Thiele et al., 2013; Mardinoglu et al., 2014). Compiling data on all reactions that have been linked to genes annotated in the human genome from various databasesas having metabolic activity is a substantial task, and important to the quality of subsequent work (Kanehisa et al., 2016; Norsigian et al., 2020). This production of annotated human genomes allowed, for the first time, metabolic networks that cover the entire human metabolic repertoire to be produced. Between 2005 and 2012, the first four human metabolic reconstructions were produced; humanCyc (Romero et al., 2004), Recon1 (Duarte et al., 2007), Edinburgh Human Metabolic Network (EHMN) (Ma et al., 2007), and Human Metabolic Reconstruction (HMR) (Agren et al., 2012). Each of these networks expanded on the previous work, including more reactions and better links between reactions and genes, allowing for improved analysis of expression data. Further improvements were made in Recon2 (Thiele et al., 2013) and HMR2 (Mardinoglu et al., 2014) and their updates (Smallbone et al., 2013; Quek et al., 2014; Sahoo et al., 2014, 2015; Swainston et al., 2016). By expanding the extent of the reaction coverage, increasing the detail of the available gene-to-reaction links, and placing more emphasis on the inclusion of good thermodynamic and stoichiometric information, better possibilities for accurate simulations were afforded; thus, these models represent significant steps forward. In addition, tools such as PathwayBooster and Path2Models have allowed the utilization of data bases such as KEGG in the automated reconstruction of new or custom built networks.

#### The Latest Human Metabolic Reconstructions Contain New Dimensions of Information

Recon3D is the latest update of the Recon family of human metabolic network reconstructions (Brunk et al., 2018). The key novel attribute of this reconstruction is that it includes information regarding protein and metabolite structures. In addition, the number of reactions included in the reconstruction has almost doubled from the previous version, to 13,453 total reactions. The inclusion of three-dimensional (3D) structural information has allowed Recon3D to be used to show that deleterious mutations map to specific areas of the genome. By using this information, improved predictions of cancer-related mutations may be made compared to previous Recon models, which include a less detailed mapping of genes. The 3D aspect of the protein and metabolite information in Recon3D has also been used to investigate the metabolic effects of various drugs; this analysis revealed that drug effect signatures often contained disparate functional domains and metabolites, indicating that many drug effects are due to compensatory downstream metabolic effects (Brunk et al., 2018). Recon3D has been constructed in such a way as to allow for integration with the AGORA platform for modeling the gut microbiome by using a consistent set of identifiers (Magúsdóttir et al., 2017). As it becomes increasingly clear that the microbiome plays an important part in human health, being able to include cometabolism by human and microbial cells is a very useful feature (Magúsdóttir et al., 2017). A global network reconstruction can then provide the starting point for the production of tissue-, cell-, or condition-specific GEMs.

### Current State-of-the-Art Applications

#### Generic Human Metabolic Reconstructions Can Be Analyzed to Gain General Metabolic Insights

A base GEM may be utilized to produce general predictions about whole human metabolism, providing useful insights into both human health and predictions of currently unknown human metabolic functions.

The various human models have been used to predict biomarkers for inborn errors of metabolism (the accuracy of previous predictions feeds into improving these models) (Kell and Goodacre, 2014). Inborn errors of metabolism (IEMs) are a collection of hereditary metabolic defects found in most of the main human metabolic pathways. These defects are usually screened for in infants by way of biofluid metabolomics (i.e., metabolomics analysis of either dried blood samples or urine). Early recognition and treatment of IEMs is very important. The current method detects specific biomarkers that have altered concentrations due to known genomic mutations. Identifying good biomarkers is key to successful early diagnosis. With the advent of the human metabolism genome-scale network model, a novel computational approach was developed that could systematically predict altered or affected metabolic biomarkers. This possible use of GEMs has the potential to extend the information that can be inferred from the data, enabling accurate diagnosis for each individual patient, further insight of hot spots in human metabolism with respect to IEMs, and discovery of novel IEMs (expanding the range of disease-associated metabolites). By making use of the GEM, gene-protein-reaction (GPR) metabolic pathways relevant to a specific genotype-phenotype pair can become more feasible, meaning that disease-specific biological insights can be derived (Shlomi et al., 2009; Sahoo et al., 2012; Mandal et al., 2018; Mussap et al., 2018).

By analyzing reactions in Recon1 that were defined as being present due to genome annotation or literature data, but that were not predicted to be active, and then adding surrounding reactions to activate them, predictions have been made about previously unknown human metabolic functions such as that of iduronic acid following glycan degradation, N-acetylglutamate in amino acid metabolism, and the human activity of gluconokinase (Rolfsson et al., 2011; Paglia et al., 2016). Such information will improve future metabolic studies both computationally and in the laboratory.

##### Reconstructions can be made specific by integrating -omics data

Once a base GEM has been selected, there are a variety of methods available to make the model specific to a particular cell type and circumstance by integrating transcriptomic, proteomic, and metabolomics data. These context-specific models are able to provide more detailed insights into human metabolism in a particular cell type, and comparison of models is particularly useful. Previous context-specific human models have produced useful insights into healthy and diseased metabolism.

A cell’s metabolic capabilities are defined by which enzymes it expresses. Transcriptomic and proteomic data provide information about the enzymes expressed under certain circumstances. Both of these data types correlate to enzyme activity, although not perfectly (Munir and McGettrick, 2015; Ibraheim et al., 2018). Several methods to prune a GEM based on expression data, mostly transcriptomics, have been developed. Although there are numerous technical differences, all seek to balance the retention of reactions known to be or likely to be present in a particular cell type, based on expression data or prior knowledge, while removing extraneous reactions. GIMME (Moreira et al., 2017), iMAT and INIT (Antonitsis et al., 2008; Vigo et al., 2019), and MBA, Fastcore, and mCADRE (Ullah et al., 2015; Archambault et al., 2017; Fitzsimmons et al., 2018) are all commonly used (Rosenbaum et al., 2008; Neman et al., 2012).

Another way to make models more context specific is to use metabolomic data collected by mass spectrometry or NMR to constrain what the model takes up or secretes to realistic values. By either measuring changes in the concentration of various metabolites in the medium over time or by comparing the relative values of metabolites at different times, the rate of uptake or secretion of a range of metabolites is determined. These rates can be applied to the model as additional constraints that will restrict the model predictions to those consistent with the metabolic dataset (Bordbar et al., 2017). These additional constraints help to predict different sets of active and inactive intracellular reactions based on extracellular data. This process may follow a protocol such as Metabotools. This protocol has been used to obtain metabolic insights into the metabolic differences between different leukemic cell lines (Aurich et al., 2015, 2016; von Bomhard et al., 2016).

Models can also be used to analyze isotope labeling data and this data can, in turn, contribute better constraints to improve the model. Cells may be fed on medium containing glucose or glutamine labeled with heavy isotopes of carbon or nitrogen. The proportions of various metabolites labeled with these heavy isotopes in cells that are sampled and analyzed at different time points after this treatment allows inferences to be made about the production of the labeled metabolites. Sholmi et al. used this technique to elucidate the differences in the TCA cycle during the cell cycle. Further information may be obtained if the cells are fractionated into different organelles before analysis (Ahn et al., 2017). For example, the subcellular localization of glutamine metabolism in cancer has been elucidated using this technique (Lee et al., 2019).

Using the model building algorithm (MBA) (Jerby et al., 2010), which generates tissue-specific models, a focused model for cancer metabolism has been created containing a core set of reactions known to be common for 60 variant cancer cell lines. Using this model and the knowledge that uncontrolled cell growth and altered metabolism are characterizing hallmarks for cancer cells, it was possible to identify two different types of drug targets (Hanahan and Weinberg, 2011; Dougherty et al., 2017). The first target type was growth-supporting genes (found via *in silico* gene deletion screens) that resulted in identification of 52 metabolic drug targets; 8 of these currently correspond to cancer therapeutics. In addition, a set of genes were identified in the healthy cell model network that were downregulated in the cancer model. By inhibiting the genes more highly expressed in cancer cells, targeting could be achieved (Dougherty et al., 2017).

More specific cancer models have also been produced. For example, a model has been created for hepatocellular carcinoma by Agren et al. (2014). They evaluated the presence of proteins in 27 patients and from that reconstructed personalized GEMs for six. These reconstructions were then used to identify anticancer drugs by observing the inhibition of reactions around each metabolite in a network and the subsequent effects on cellular growth within the models. By conceptualizing drugs as structural analogs to metabolites, and so capable of interfering with target enzymes and enzymatic activity, 101 antimetabolites were predicted as possible drug targets (Agren et al., 2014). Similar approaches have been applied to breast cancer undergoing epithelial-to-mesenchymal transition in order to identify targets to reduce this pro-metastatic process (Halldorsson et al., 2017).

#### Comparing Models of Cells in Different Circumstances Can Produce Useful Insights Into Metabolism

Many constraint-based metabolic analyses have historically relied upon an objective function, which is defined as a metabolic objective of a cell; flux through this reaction is either maximized or minimized to compute the flux state (i.e., pathway usage) across the entire network. For metabolic states that do not have as well-defined objective functions as cancer does (i.e., gross cellular growth), algorithms that are able to create tissue- or cell-specific models without a specific objective function are needed. An algorithm often used for this purpose is the metabolic transformation algorithm (MTA) (Yizhak et al., 2013), an algorithm that uses GEMs to predict genetic perturbations that are able to shift a diseased metabolic state toward a healthy one. This algorithm has been used to determine reactions capable of shifting “old” muscle into “young” (providing potential targets that can help reducing metabolic shifts related to aging) and to determine key reactions that, when removed from a GEM modeling Alzheimer’s disease, resulted in a network reconstruction more similar to that of a healthy state (Stempler et al., 2014; Wone et al., 2018).

Obesity has been addressed through the use of the human metabolic reconstruction by identifying pathways implicated in the disease process. As with many diseases, pinpointing a specific genetic or environmental marker as a cause for obesity, making the determination of progression, and deciding on a “treatment” a difficult task. Using the HMR and transcriptomic data from both healthy and obese individuals, a GEM with the objective function defined as acetyl-CoA production and formation of lipid droplets was produced. Through this analysis, two possible drug targets were identified by considering reactions with significantly changed flux values, and a potential biomarker for obesity was identified through reporter metabolites, which is an algorithm allowing the analysis of transcriptomic data in the light of the metabolic network structure to predict highly affected metabolites (Bordbar et al., 2011; Väremo et al., 2013; Levian et al., 2014; Dougherty et al., 2017).

Drug toxicity levels and side effects over both short and long periods of usage can also be identified in an easier and more cost-efficient manner using GEMs. It is possible to make predictions on system-wide perturbations using previously determined information on how protein structural analysis can be used to determine off-target binding of drugs, in combination with metabolic networks, as was done by Chang et al. (2010).

#### Metabolic Models Can Be Used to Uncover Changes Over Time

Biological systems often change dynamically over time. Analyzing how these changes occur can be challenging but is being addressed through the integration of time-course experimental data. One approach, dynamic flux balance analysis (dFBA) {ref 10.1016/j.celrep.2017.07.04}, integrates time-course measurements of the major inputs and outputs of the system to provide more accurate flux predictions. dFBA provides a continuous prediction based on these changing inputs and outputs (e.g., end products of pathways). This method has been applied to murine embryonic stem cells and revealed changes to mitochondrial metabolism and one carbon metabolism during priming (Shen et al., 2019). more recently, time-course -omic measurements have been integrated with metabolic models. One such method, unsteady-state flux balance analysis (uFBA), integrates absolutely quantified time-course metabolomic data to model cellular dynamics. uFBA was used to explore how temporal dynamics impact the cellular metabolism of stored red blood cells, which led to the proposal of better storage solutions that could potentially increase the storage time and quality of this key medical product. Such methods may be applied to other cell types and phenotypes in the future where dynamics play a key role. In MSCs, for example, key metabolic shifts that occur during trilineage differentiation may be examined and compared as a function of time.

#### A Metabolic Model of MSCs Has Already Predicted Better Ways to Expand MSC Cultures

A GEM of MSCs, iMSC1255, was recently created to improve understanding of the function of MSC metabolism. This model is based upon publicly available transcriptomic data sets from proliferating, early passage bone marrow MSCs. The data was used with the mCADRE algorithm to generate a tissue-specific version of the global human model Recon1, which was then manually curated by comparison to proteomic data and the literature to ensure that all desirable reactions were included to account for known MSC metabolic functions (Wang et al., 2012). Further metabolic constraints were added based on the composition of the commonly used medium alpha MEM, which meant that the modeled cells were able to take up metabolites known to be available in alpha MEM. This model was compared to previous models created with the same algorithm for adipose, bone marrow, and blood (using tissue-based, rather than cell-based, models). These previous models were shown to be less specific than iMSC1255. iMSC1255 was also shown to be able to produce amino acid uptake and secretion and growth rate predictions consistent with data available in the literature (Fouladiha et al., 2015).

iMSC1255 has subsequently been used to computationally predict metabolic interventions to optimize proliferation and chondrogenic differentiation of MSCs. By analyzing the maximum growth rate predicted FBA with and without allowing the uptake of a range of nutrients, it was proposed that supplementing the MSC medium with the phospholipids phosphoethanolamine and phosphoserine would improve proliferation. This was confirmed experimentally (Fouladiha et al., 2018). This paper also describes how MTA was used along with transcriptomic data from chondrocytes to determine likely metabolic changes during chondrocyte differentiation. This analysis suggested that mitochondrial transport reactions are key to chondrocyte differentiation, a finding that has yet to be experimentally confirmed. Further, the authors also examined the effects of hypoxia on proliferating MSCs by assessing the range of possible metabolic activities when the models use different levels of oxygen and glucose availability. The predicted metabolic changes to lactate and glucose uptake and secretion, G6P isomerase, and pyruvate transport were generally correct, with the exception of superoxide dismutase, according to the literature (Fouladiha et al., 2018). This study showed that a model of MSC metabolism can provide useful insights into their proliferation under different circumstances. This will allow the optimization of MSC growth that may be useful for large scale production of MSC-based therapeutics. Follow-up work has begun to examine the potential of GEMs to predict changes necessary for successful differentiation (Fouladiha et al., 2018). By expanding upon such techniques, including by using models constrained with metabolic data from differentiating cells, this can be built upon.

## Looking Ahead

Since 2007, when the very first global GEM for humans was reconstructed (Duarte et al., 2007), researchers have been exploring the clinical application possibilities of GEMs. Some of the possible ways GEMs can be of use in furthering the clinical application of cell-based medicine include: (1) trials with *in silico* metabolic engineering (gene knock-outs and knock-ins); (2) identifying biomarkers of diseases; (3) predicting drug targets and therapeutic windows; and (4) optimization of cellular functions without the cost of wet lab experimentation. Some success has already been reported, as mentioned in section “Current State-of-the-Art Applications.”

### Manipulation of the Metabolic State

Building on the various uses of existing cell- or tissue-specific human GEMs and the most up-to-date version of the human reconstruction (Brunk et al., 2018), the potential use of GEMs to explore methods to maintain or manipulate a desired metabolic state for hMSCs (in order to provide a specific function or desired effect) has been the subject of work done by Fouladiha et al. They demonstrated the potential of GEMs to gain insight into how MSCs may be manipulated by means of nutrient supplementation (Fouladiha et al., 2018). By adding nutrients into the growth medium or manipulating oxygen concentration *in silico*, the number of experiments needed to optimize growth conditions may be reduced. This may be an interesting avenue to explore for each cell type and cell state, since different responses may be observed. Such testing is more feasible to explore *in silico* than *in vitro*. Even though time and money will have to be spent on reconstruction of the GEMs themselves, the savings in experimental time and material costs afforded by the use of validated reconstructions will likely outweigh these costs. Promising *in silico* outcomes can then be taken further, being validated or explored *in vitro* and later *in vivo.* Positive results would further validate the models and perhaps further that particular avenue of cell-based medicine or that particular use of the cells in regenerative medicine.

### Exploration of Metabolic Differences

One potential use for GEMs would be to explore the different metabolic capacities of hMSCs from different sources. There have been some reports, albeit limited, explaining possible differences in the proliferative, differentiation, and immunomodulatory abilities of hMSCs isolated from various tissues (Liu et al., 2007; Hass et al., 2011; Secunda et al., 2015; Tachida et al., 2015). As hMSCs are isolated from disparate microenvironments, some with large differences in their surroundings, their optimal survival conditions and, possibly, utilization potential may differ. For example, by comparing models created using transcriptomics data from a study of MSCs from various sources such as ArrayExpress (EMBL-EBI, 2014; Athar et al., 2019) and then subjecting them to comparative flux analysis, different metabolic patterns may be discovered and linked to previously reported functional differences, such as the ability to form hematopoietic cells. Results could be verified or supplemented with data from independent proteomics experiments such as can be found via PRIDE Archive (Billing et al., 2016).

The creation and study of GEMs of MSCs undergoing each of the three classical differentiation (adipo-, osteo- and chondrogenic) could be approached in various ways. uFBA could be used as a framework to examine MSC metabolomics data collected at different timepoints during each of the differentiation lineages (Bordbar et al., 2017). The study of other stem cell types over time has already provided useful insights into differentiation from a transcriptional viewpoint. uFBA would allow a better understanding of the metabolic changes to be reached (Bordbar et al., 2017).

### Experimental Cost Reduction via *in silico* Result Prediction

As expansion and differentiation of MSCs is a time-consuming and potentially expensive process, it would be desirable to be able to predict in advance the success of cells from a particular donor. For example, a signature pattern of gene features could be tested before donation. To this end, a model of successful and unsuccessful cells created with Recon3D could be used to analyze the genes relevant to an ideally differentiated model *in silico* (Strober et al., 2019). Single and combined gene deletion to find genes essential to each form of differentiation would be useful as a secondary way of finding a genetic signature for successful differentiation. This process could provide a means of reducing the necessary number of *in vitro* analyses.

It would also be desirable to confirm adequate differentiation by measuring a few metabolites before attempting implantation. The reporter metabolites algorithm (Çakır, 2015; Schultz and Qutub, 2016) applied to a model of well- differentiated MSCs would determine exometabolomic biomarkers that are indicative of successful levels of differentiation.

### Multi-Cell Models

Community models or multi-cell models are another avenue to be explored as a potential use for GEMs to enhance the use of hMSCs in regenerative medicine. These models can provide insight into the metabolic functions of possible interacting organisms or the various cell types residing within the same organism (Levy and Borenstein, 2013; Dougherty et al., 2017). Given that hMSCs are intended to be integrated into a host system for clinical application (Han et al., 2012; Levy and Borenstein, 2013; Burke et al., 2016; Archambault et al., 2017; Goldberg et al., 2017; Moreira et al., 2017; Cunningham et al., 2018; Hu et al., 2018; Ibraheim et al., 2018; Solarte et al., 2018), this could provide useful insights.

Recently, more attention has been given to the hMSC secretome and its possible therapeutic effect; this might be well explored through the use of multi-cellular GEMs. The secretome of hMSCs might be manipulated via some of the previously mentioned methods, and the effect of composition changes on the targeted organism, cell, and/or environment observed. This could help to find novel directions in which to expand the use of hMSCs in regenerative medicine.

### Age Related Exploration

Yet another aspect that could more easily be explored through the use of GEMs covering hMSCs is the effect of donor age. This is not the same as cell senescence, but effects due to age have been observed in hMSCs lines through *in vitro* experiments (Choudhery et al., 2014; Narbonne, 2018). This appears in the way that cells are able to perform with regard to proliferation speed, differentiation ability, and immunomodulation. There have been numerous attempts to return functionality to stem cells from aged donors, with some degree of success (Neves et al., 2017). However, with the application of GEMs, the significant changes and the reasons behind them may be more systematically documented and attempts to return function performed in a more cost-effective and time-saving manner.

Overall, the use of GEMs to further the use of hMSCs in regenerative medicine is increasing, but, as of yet, is a relatively unexplored avenue that holds a lot of promise. We anticipate that *in silico* metabolic modeling will help to elucidate the differentiation process of hMSCs, ultimately providing crucial insights into novel therapies in the field of regenerative medicine.

## Author Contributions

ÞS and SM were the main authors of the manuscript. All additional authors (ÓR, JY, and ÓS) also contributed to the writing and editing of the manuscript.

## Conflict of Interest

The authors declare that the research was conducted in the absence of any commercial or financial relationships that could be construed as a potential conflict of interest.
